# Enhanced Visible-Light Photocatalytic Activity of Bismuth Ferrite Hollow Spheres Synthesized via Evaporation-Induced Self-Assembly

**DOI:** 10.3390/molecules29153592

**Published:** 2024-07-30

**Authors:** Thomas Cadenbach, Valeria Sanchez, Karla Vizuete, Alexis Debut, Carlos Reinoso, Maria J. Benitez

**Affiliations:** 1 Departamento de Ingeniería Ambiental, Instituto de Energía y Materiales, Colegio Politécnico de Ciencias e Ingenierias, Universidad San Francisco de Quito, Quito 170901, Ecuador; 2 Departamento de Física, Facultad de Ciencias, Escuela Politécnica Nacional, Ladrón de Guevara E11-253, Quito 170517, Ecuador; 3 Centro de Nanociencia y Nanotecnología, Universidad de las Fuerzas Armadas ESPE, Av. Gral. Rumiñahui s/n, Sangolquí 171523, Ecuador; 4 School of Physical Sciences and Nanotechnology, Yachay Tech University, Hda. San José s/n y Proyecto Yachay, Urcuqui 100115, Ecuador

**Keywords:** nanostructured materials, semiconductors, catalysis, hollow spheres, self-assembly

## Abstract

Semiconductor hollow spheres have garnered significant attention in recent years due to their unique structural properties and enhanced surface area, which are advantageous for various applications in catalysis, energy storage, and sensing. The present study explores the surfactant-assisted synthesis of bismuth ferrite (BiFeO_3_) hollow spheres, emphasizing their enhanced visible-light photocatalytic activity. Utilizing a novel, facile, two-step evaporation-induced self-assembly (EISA) approach, monodisperse BiFeO_3_ hollow spheres were synthesized with a narrow particle size distribution. The synthesis involved Bi/Fe citrate complexes as precursors and the triblock copolymer Pluronic P123 as a soft template. The BiFeO_3_ hollow spheres demonstrated outstanding photocatalytic performance in degrading the emerging pollutants Rhodamine B and metronidazole under visible-light irradiation (100% degradation of Rhodamine B in <140 min and of metronidazole in 240 min). The active species in the photocatalytic process were identified through trapping experiments, providing crucial insights into the mechanisms and efficiency of semiconductor hollow spheres. The findings suggest that the unique structural features of BiFeO_3_ hollow spheres, combined with their excellent optical properties, make them promising candidates for photocatalytic applications.

## 1. Introduction

Water pollution is a pressing global issue that poses a significant threat to both human health and the environment [1]. Contaminants like organic dyes, heavy metals, pesticides, and pharmaceutical residues degrade aquatic ecosystems and compromise clean water resources [2,3]. Thus, there is an urgent need for effective and environmentally friendly techniques to tackle this challenge and restore the purity of water sources.

Visible-light photocatalysis, powered by semiconductor materials, has emerged as a promising technology for combating water pollution [4,5,6,7,8]. This approach utilizes semiconductors to drive photochemical reactions under illumination with visible light, leading to the degradation and removal of a wide range of pollutants [7,8]. Unlike traditional treatment methods that rely on chemical reagents, high energy inputs, or expensive equipment, visible-light semiconductor photocatalysis harnesses the visible-light fraction of the solar spectrum and operates under mild reaction conditions, minimizing the generation of secondary pollutants and reducing energy consumption. This development allows the utilization of abundant and renewable solar energy for water purification, making the process more sustainable and cost-effective. The unique structural characteristics and properties of semiconductor hollow spheres offer remarkable advantages in photocatalysis, opening new avenues for sustainable water remediation [9,10,11,12,13,14,15]. Semiconductor hollow spheres possess a hollow interior and a porous outer shell, providing a large surface area and abundant active sites for photocatalytic reactions. In addition to their large surface-to-volume ratio, semiconductor hollow spheres offer several advantages over conventional photocatalysts. For instance, hollow spheres exhibit improved light-harvesting capabilities, as they can trap and scatter incident light within the hollow cavity, maximizing light utilization and enhancing the overall photocatalytic performance. Additionally, the unique hollow structure provides a large internal volume, which can accommodate a higher loading of photocatalytic material [9,10,11,12,13,14,15]. This increased material loading results in enhanced photocatalytic activity and improved efficiency in pollutant degradation. Furthermore, the overall efficiency is also increased by the combination of their high crystallinity and large surface area. The unique architecture facilitates the efficient diffusion and transfer of reactants and products, which leads to faster reaction kinetics.

The synthesis of nano- and micro-structured hollow spheres can be achieved by various templating techniques, hydrothermal approaches, and solvothermal methods [9,11,12,14,15,16,17,18,19,20]. Sacrificial hard templates using prefabricated carbon or silica nanospheres often yield monodisperse and well-defined hollow spheres [18]. However, apart from the additional reagents that are required for the synthesis, the pre-synthesis of hard templates is often time-, work- and cost-intensive. Hydrothermal and solvothermal techniques, on the other hand, often allow for single-step syntheses and offer further advantages such as scalability and environmentally friendliness, as water is mostly used as a solvent. However, we [9] and others [19,21,22,23] have demonstrated recently that hydrothermal synthesis often lacks precise control over the morphology of the produced hollow spheres. Additionally, it typically requires prolonged reaction times, as well as high-pressure and high-temperature conditions, restricting the range of materials that can be used in the synthesis, and it often requires specialized equipment and energy-intensive procedures [19].

During recent years, the self-assembly of soft templates such as the poly(ethylene oxide)-poly(propylene oxide)-poly(ethylene oxide) triblock copolymer Pluronic P123 has been successfully applied in the synthesis of nanoporous inorganic materials [20,24,25,26,27,28,29,30,31]. In this context, the evaporation-induced self-assembly (EISA) process has been used to prepare mesoporous metal oxides [25,32,33,34,35,36]. EISA offers advantages such as simplicity, tunability, scalability, uniformity, versatility, and control over porosity. The EISA approach initially requires the preparation of a homogeneous solution of the inorganic precursors and the soft template, followed by an evaporation of the solvents. This induces the self-assembly of the soft templates into micelles and other soft meso-structures, as well as the self-assembly of the inorganic precursors around the soft templates. The application of the EISA approach for the synthesis of porous and well-structured perovskites is largely underdeveloped due to the poor solubility of the inorganic materials, leading to heterogeneous mixtures and secondary phases in the isolated materials. Furthermore, the high calcination temperatures required for calcination in perovskite syntheses often lead to a collapse of the organic templates and, thus, to the destruction of the morphology, whereas low calcination temperatures lead to amorphous and secondary products [35]. To obtain more soluble organometallic precursors and to facilitate the EISA approach, researchers have applied organic chelating agents such as citric acid, urea, or acetic acid in the syntheses [35,36].

The effectiveness of hollow spheres as visible-light photocatalysts hinges on the choice of semiconducting materials, with bismuth ferrite (BiFeO_3_) drawing significant attention for its unique properties [37,38,39,40,41,42,43]. BiFeO_3_ exhibits excellent chemical stability, a narrow band gap in the visible-light range (2.0–2.8 eV), a relatively slow electron–hole recombination rate, and multiferroic properties, making it an ideal candidate for visible-light photocatalysis and electronic applications such as data storage media, multi-state memories, and quantum electromagnets. However, the size, morphology, crystallinity, and purity of BiFeO_3_ significantly influence its optical properties and photocatalytic performance. Synthesizing nanosized BiFeO_3_ with a high surface area often leads to the formation of oxygen defects and surface constraints, resulting in reduced photocatalytic activity. Additionally, the volatilization of bismuth during calcination can lead to the formation of secondary phases like Bi_2_Fe_4_O_9_ and Bi_2_O_3_, making the synthesis of pure single-phase BiFeO_3_ challenging, and necessitating precise control over the synthesis conditions [40,41,42].

Despite these challenges, a few methods for synthesizing single-phase BiFeO_3_ hollow spheres have been reported in the literature [18,44,45,46]. These include a hard templating approach using carbon nanospheres as templates, resulting in photocatalytically active hollow spheres [45]. Passionfruit-like Bi@BiFe-glycolate hollow spheres have also been synthesized as precursors for Bi_2_O_3_/BiFeO_3_ composite hollow spheres, demonstrating enhanced photocatalytic activities in organic pollutant degradation [18]. Additionally, our group reported the first synthesis of single-phase BiFeO_3_ hollow spheres using a facile hydrothermal method, yielding hollow spheres with exceptionally high photocatalytic efficiencies [9]. However, the purity and morphology of the hollow spheres were found to be sensitive to synthetic conditions, resulting in a wide size distribution ranging from 200 nm to 2 µm.

In the present work, we report the successful synthesis of monodisperse single-phase BiFeO_3_ hollow spheres with a narrow particle size distribution by employing a facile two-step evaporation-induced self-assembly approach. Prefabricated Bi/Fe citrate complexes were used as precursors, and the triblock copolymer Pluronic P123 was utilized as the soft template in a 2-methoxyethanol/HNO_3_ solvent mixture. The resulting materials were then applied in photodegradation reactions of the emerging pollutants Rhodamine B and metronidazole.

## 2. Results and Discussion

**Figure 1:**
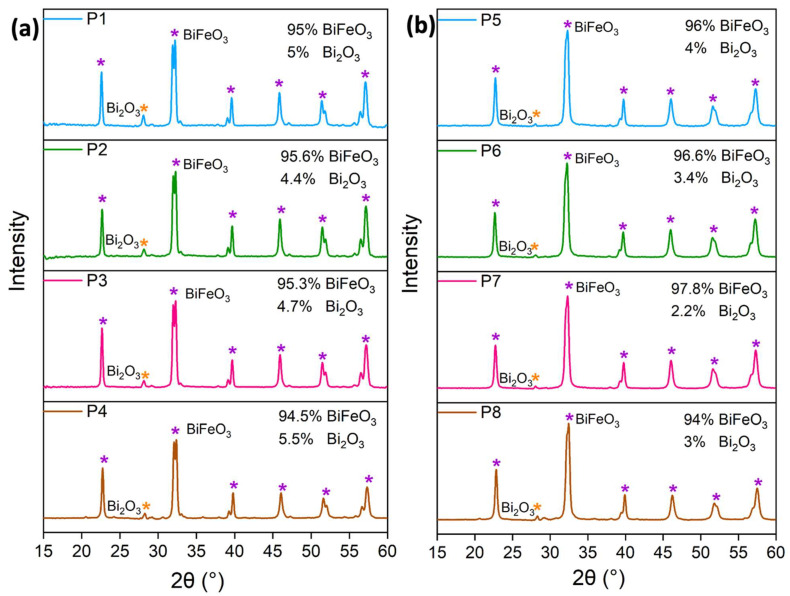
(**a**) X-ray diffractograms of BiFeO_3_ samples with different Pluronic P123 contents. (**b**) X-ray diffractograms of Gd_0.05_Bi_0.95_FeO_3_ samples with different Pluronic P123 contents.

At first, we followed a modified synthesis described by Wang et al., using the well-known triblock copolymer Pluronic P123 as the structure-directing agent and citric acid as a complexing agent [47]. In a first approach, a solution of the hydrated bismuth and ferric nitrates as well as citric acid in water was added dropwise to a solution of P123 in a mixture of ethanol and H_2_O. It should be noted that Bi^3+^ and Fe^3+^ form citric acid–metal complexes in solution via the chelating effect [48,49,50]. The impact of different P123 concentrations was tested, and the XRD patterns are shown in Figure 1a, while the sample indexation is defined in Table 1.

All samples were highly crystalline and corresponded primarily to BiFeO_3_ in a rhombohedral structure (space group R3c, JCPDS card No/86-1518), which can be clearly identified by the typical peak splitting of the (104) and (110) peaks. In addition to the BiFeO_3_ main phase, slight amounts of Bi_2_O_3_ (JCPDS card No/27-0050) as a minor byproduct can be assigned to the remaining peaks in the diffractograms. The increase in the concentration of P123 from 2.72 mM to 4.6 mM did not affect the intensity of the diffraction peaks and, thus, it did not influence the crystallinity or phase purity of the products. In all cases, a semi-quantitative analysis with the DIFRACC.EVA software suite version 4.3.1.2 showed approximately 4.4–5.5% impurities in the form of Bi_2_O_3_. While such minor impurities can be washed away by glacial acetic acid or diluted nitric acid without affecting the morphologies [9,51], we decided to reduce additional post-modification steps by aiming for a direct synthesis of phase-pure and highly crystalline material. Thus, we decided to modify the synthesis and substitute 5% bismuth nitrate with 5% gadolinium nitrate while keeping the remaining synthesis procedure identical to the undoped synthesis. It has been shown that the doping of bismuth ferrite with gadolinium not only eliminates unwanted impurity phases such as Bi_2_O_3_ but also results in photocatalysts with increased efficiency [52,53]. The XRD patterns of the doped samples are shown in Figure 1b. The doping on the bismuth site resulted in an expected shift of the (104) and (110) Bragg peaks towards higher 2θ, as well as a decrease in the splitting intensity of the same peaks. This can be attributed to a slight distortion of the rhombohedral structure upon incorporation of the smaller gadolinium ion [52,53,54]. However, the diffractograms of the doped samples also display minor impurities in the form of Bi_2_O_3_ between 2.2 and 4%.

The actual doping concentration was determined by an inductively coupled plasma optical emission spectroscopy analysis (ICP-OES). Here, all samples were characterized by dopant concentrations very close to the theoretical calculated concentrations (see Table 1).

**Figure 2:**
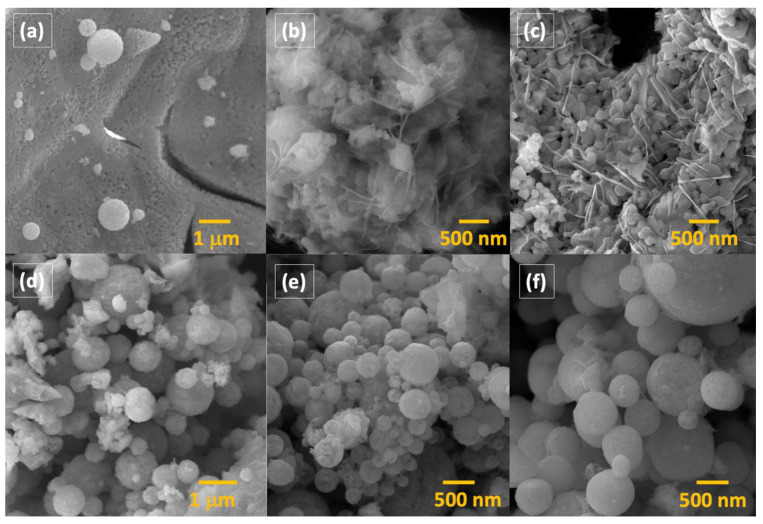
SEM images of BiFeO_3_ samples: (**a**) hollow spheres on bulk material in P1; (**b**) bulk material in P2; (**c**) bulk material in P4; (**d**) hollow spheres in P5; (**e**) hollow spheres in P6; (**f**) hollow spheres in P8.

The morphologies of the synthesized samples were determined by scanning electron microscope images, which are shown in Figure 2. The scanning electron microscopy analysis indicated that the synthesis process resulted in the formation of hollow spheres across all samples. These hollow spheres had diameters ranging from 50 nanometers (nm) to 2 micrometers (µm). Additionally, the surfaces of these spheres were rough, consisting of smaller particles. In addition to the presence of these hollow spheres, most of the material comprised irregularly shaped, bulk-sized material. Interestingly, the gadolinium-doped samples appeared to have a higher concentration of hollow spheres, yet they also predominantly exhibited irregular bulk material. In both cases, i.e., doped and undoped samples, an increase in the P123 concentration did not result in significant changes in the morphology of the samples.

The elemental composition of the hollow spheres and of the bulk material appears to be identical, as it shows a uniform distribution of Bi, Fe, and O at the expected ratio of 1:1:3, as determined by energy-dispersive spectra measurements (shown in Figure S1 in the Supplementary Materials).

**Figure 3:**
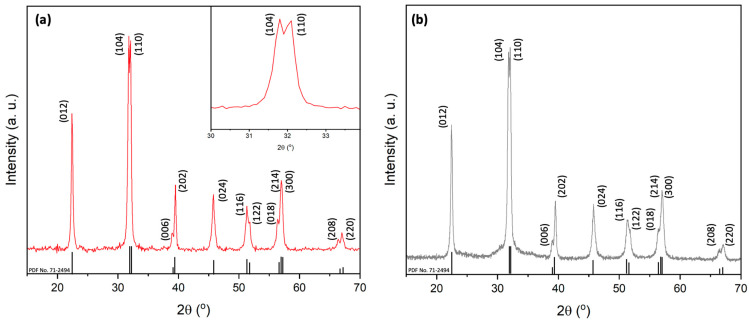
(**a**) X-ray diffractograms of BiFeO_3_ sample P9 before photocatalysis. (**b**) X-ray diffractograms of BiFeO_3_ sample P9 after 4 photocatalytic degradation cycles.

As outlined in the introduction, the general difficulty and the desired goal in BiFeO_3_ synthesis is to achieve a phase-pure material with the anticipated morphology. In this context, it has been shown that bismuth nitrate hydrolyzes rapidly, which results in the formation of multinuclear bismuth species such as [Bi_6_O_4_(OH)_4_(NO_3_)_5_(H_2_O)](NO_3_) and [Bi_6_O_4_(OH)_4_(NO_3_)_6_(H_2_O)_2_]·H_2_O [55]. It should be noted that these species even form in H_2_O solutions previously acidified with nitric acid. These hexanuclear bismuth oxido clusters can further react and aggregate to poorly soluble [Bi_38_O_45_]^24+^ clusters. The final hydrolysis results in the formation of Bi_2_O_3_. To prevent hydrolysis of bismuth nitrate and the formation of Bi_2_O_3_ as well as poorly soluble intermediates during the soft templating process, we synthesized bismuth and iron citrate metal complexes prior to the soft templating. Chelating compounds play a crucial role in controlling the shape and size of particles, as well as the decomposition temperatures of the precursor, which will then influence the crystallinity and purity [48,49,50]. The importance of citric acid as a chelating agent has been previously reported in the synthesis of BiFeO_3_ in general [40,56], and in the formation of perovskite hollow spheres [47]. In general, hydroxycarboxylic acids such as citric acid and tartaric acid form polybasic acid chelates with metal ion centers and can undergo esterification reactions when heated [48,49,50]. Furthermore, bismuth and iron centers can be bridged by the acid ligands, resulting in heterometallic polynuclear coordination polymers, which can be further stabilized by hydrogen bonding between the ligands and the solvent [48,49,50]. The decomposition of these polynuclear species results in the formation the final product, i.e., BiFeO_3_. In the present study, we first added the metal nitrates along with the complexing agent citric acid to 2-methoxyethanol. Secondly, after stirring the solution for 12 h, the metal complex precursor was isolated by drying the reaction mixture. It should be noted that these Bi and Fe citrate complexes have been described in detail previously [48,49,50]. The isolated precursors were then dissolved in a solvent mixture containing 2-methoxyethanol, water, and 2 M nitric acid. The homogenous precursor solution was then added to a solution of P123. After solvent evaporation, the resulting powder was calcined, and the XRD diffractogram is shown in Figure 3a. The diffraction pattern of the resulting product (P9) shows the formation of a rhombohedral BiFeO_3_ structure with an R3c space group (JCPDS No. 86-1518), as indicated by the clearly separated (104) and (110) diffraction peaks. No additional peaks for secondary impurities or other phases, such as Fe_2_O_3_, Bi_2_O_3_, or Bi_2_Fe_4_O_9_, were observed in any case. This demonstrates the effectiveness of the synthesis method in producing phase-pure BiFeO_3_ material.

**Figure 4:**
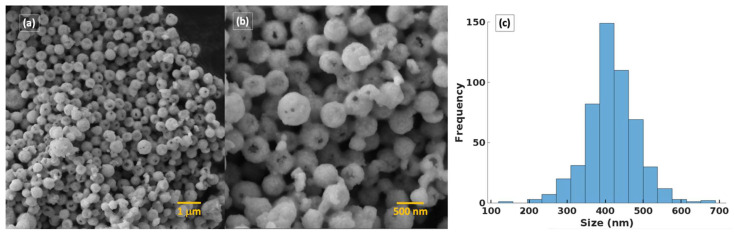
(**a**,**b**) SEM images of BiFeO_3_ hollow spheres (P9). (**c**) Particle size distribution.

Figure 4 displays the scanning electron microscopy images of the sample synthesized by the EISA method. The SEM analysis reveals that the morphology is characterized by monodisperse hollow spheres with a diameter ranging from 200 to 650 nm. The particle size distribution of P9 was estimated from the SEM image analysis by using ImageJ software version 1.54j. The histogram follows a log-normal distribution function, with a peak at approximately 400 nm indicating the average sphere size (see Figure 4). The hollow spheres are composed of smaller crystallites that are visible on the surface and contribute to an overall rough surface. Open, incomplete spheres reveal the hollow nature of the spheres. The wall thickness of these spheres ranges from 40 to 70 nm.

The surface area of the hollow spheres was determined by nitrogen sorption using Brunauer–Emmet–Teller (BET) analysis (see Figure S2). The BET plot shows a typical type IV class isotherm with a type H4 hysteresis loop, which is characteristic of mesoporous materials. The obtained BET surface area of the BiFeO_3_ hollow spheres was 39 m^2^ g^−^^1^. The pore sizes are distributed widely, with two maxima at 3.8 nm and 22.8 nm.

**Figure 5:**
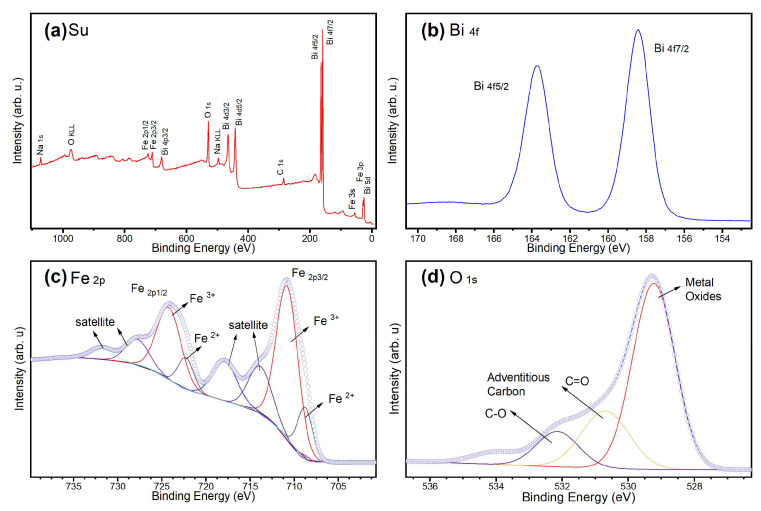
Core-level analysis of BiFeO_3_ by XPS: (**a**) Survey spectrum showing the main chemical components of the sample. (**b**) High-resolution spectrum of the Bi 4f electronic level, indicating a +3 oxidation state. (**c**) High-resolution spectrum of the Fe 2p level, with deconvolution showing predominant Fe^3+^ and minor Fe^2+^ states. (**d**) High-resolution spectrum of the O 1s core level, displaying peaks corresponding to metal oxides and contamination from adventitious carbon.

The survey XPS analysis of BiFeO_3_, as shown in Figure 5a, clearly identified the presence of Bi, O, and Fe bands. The detected carbon was attributed to adventitious carbon, commonly found on surfaces exposed to air [57]. The O 1s core-level spectrum displays a main peak at 529 eV, corresponding to metal–oxygen bonds (Bi-O and Fe-O), as presented in Figure 5b. Additional peaks at 531.5 eV and 532.5 eV were assigned to adventitious carbon species (C=O and C-O, respectively) [58]. The spin–orbit splitting of the Bi 4f electronic level shows Δ = 5.3 eV, as expected. The Bi 4f7/2 peak centered at 158.7 eV is associated with the +3 oxidation state of Bi [58,59,60]. In the high-resolution XPS spectrum of the Fe 2p electronic level (Figure 5), Fe^3+^ was identified as the predominant oxidation state, with minor contributions from Fe^2+^. The main Fe^3+^ peak is observed at 710.8 eV, with a satellite peak at 717.9 eV, indicating an 8 eV separation. For Fe^2+^, the main peak is at 708.8 eV, and the satellite peak is at 713.9 eV, showing a 6 eV separation. The spin–orbit splitting between the Fe 2p3/2 and Fe 2p1/2 peaks is approximately 13 eV, consistent with the expected values for iron. In the Fe 2p3/2 region, peaks are observed at 722 eV for Fe^2+^ and 723.8 eV for Fe^3+^, with corresponding satellite peaks at 727.8 eV and 732 eV, respectively [58,60]. These results confirm that Fe^3+^ is the dominant species, with Fe^2+^ present in smaller proportions. The peak separations align with typical values for these oxidation states, providing a clear understanding of the iron chemistry and electronic structure within the material. The analysis confirms that the composition of BiFeO_3_ matches the expected chemical environment, as evidenced by the XPS data.

**Figure 6:**
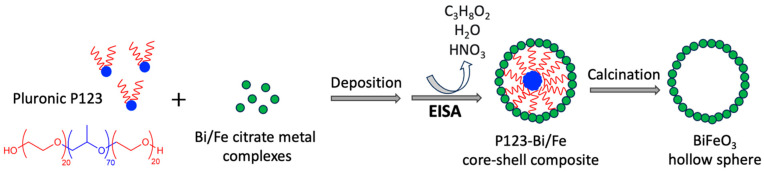
Schematic illustration of the formation process of BiFeO_3_ hollow spheres.

A possible mechanism for the formation of BiFeO_3_ hollow spheres is based on a multi-component solvent evaporation-induced self-assembly (EISA) of the block copolymer P123 (see Figure 6) [34]. It has been shown that organic chelating reagents such as acetic acid or citric acid act as additional macroscale templates enabling the formation of structures above the mesoscale (2–50 nm) [34]. In the current study, citric acid functioned as a chelating agent, binding to bismuth and iron to form metal–citrate complexes. This helps regulate the hydrolysis of metal ions and prevents premature precipitation. The block copolymer P123 is amphiphilic, with hydrophobic and hydrophilic segments, allowing it to form micelles in the solvent. When the precursor solution is subjected to controlled evaporation, the concentration of the block copolymer P123 and metal–citrate complexes increases, which causes P123 to self-assemble into micelles. The metal–citrate complexes start to interact with the micelles. Here, the stability of the template mesophase during synthesis is believed to be maintained through interactions between the metal precursor and the polar polyethylene oxide (PEO) blocks in the surfactant P123 [20,61]. Specifically, hydrogen bonding between metal complexes and the PEO groups plays a crucial role in preserving the mesophase structure. This interaction ensures that the organized arrangement of the template is retained throughout the synthesis process, leading to the successful formation of the desired nanostructures [20,61]. Further evaporation of the solvent leads to the organization of these micelles into a regular pattern and a mesostructured template. Calcination is performed to remove the block copolymer template and to crystallize the BiFeO_3_. During this process, the polymer burns off, leaving behind hollow structures where the micelles were originally located. The calcination also promotes the decomposition of the metal–citrate complexes and the formation of the metal oxides, with the formation of BiFeO_3_ as the end result. This proposed mechanism highlights the crucial role of each component in the synthesis process, from the correct choice of solvent, to the formation of metal–citrate complexes and micelles, to the final calcination step that yields the hollow BiFeO_3_.

The band gap energy of the isolated BiFeO_3_ hollow spheres was determined by the Kubelka–Munk method using the measured diffuse reflectance UV–Vis spectrum (see Figure S3 in the Supplementary Materials). The UV–Vis spectrum displays an absorption band edge at 555–565 nm, demonstrating significant visible-light absorption capabilities, which underlines the potential of these hollow spheres to be ideal candidates for the intended visible-light advanced oxidation degradation reactions. The Tauc plot, i.e., the tangent line in the plot of the square root of the Kubelka–Munk function versus photon energy, reveals a band gap energy of 2.12 eV, which is redshifted compared to bulk BiFeO_3_ and comparable to the band gap energy found in the BiFeO_3_ hollow spheres described earlier [9,51,54]. It should be noted that band gap energies are usually difficult to compare, since oxygen vacancies, phase purity, and the size and shape of the BiFeO_3_ particles have a significant impact on the band gap. For example, symmetry breaking in materials with high surface areas and strain effects can cause a redshift in the band gap [62,63,64,65].

**Figure 7:**
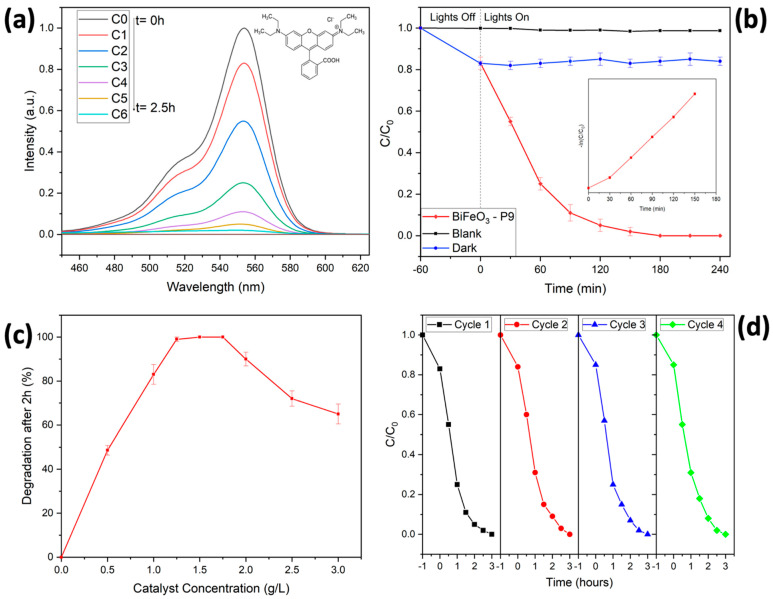
Photocatalytic degradation of Rhodamine B using BiFeO_3_ (sample P9): (**a**) UV-Vis spectra of Rhodamine B during the degradation reaction. (**b**) Removal of Rhodamine B as a function of irradiation time. (**c**) Influence of catalyst concentration on degradation. (**d**) Reuse of the BiFeO_3_ hollow spheres in consecutive degradation reactions.

The photocatalytic properties of the BiFeO_3_ hollow spheres under visible-light illumination were assessed using Rhodamine B, a common organic pollutant in the dye industry. The degradation efficiency was evaluated by plotting the ratio of C/C0 over time, where C0 represents the initial maximum intensity and C represents the maximum intensity as a function of irradiation time (see Figure 7). It is important to note that Rhodamine B is extremely stable under visible-light irradiation in the absence of a photocatalyst. Consequently, no significant degradation of the dye was observed even after 4 h of irradiation, based on the intensity of the maximum absorbance peak of Rhodamine B at 553 nm (see Figure 7b). Equilibrium between adsorption and desorption of Rhodamine B on the catalyst surface was reached in about 1 h. Approximately 17% of the dye’s initial concentration was adsorbed, which could be easily desorbed using a solvent mixture of 2-methoxyethanol and H_2_O [9,18].

The adsorption capability of the synthesized BiFeO_3_ hollow spheres was slightly enhanced compared to previously reported values, which can be explained by the smaller particle size and consequently higher surface area [37,43]. Visible-light irradiation (λ = 427 nm, 440 nm) of the reaction mixture resulted in the degradation of Rhodamine B, as evidenced by a decrease in the maximum absorbance peak at 553 nm. In approximately 140 min, Rhodamine B was completely degraded. After washing the isolated catalyst with a mixture of 2-methoxyethanol and H_2_O, no dye was detected in the washing solution, confirming the total degradation of Rhodamine B within that timeframe. The degradation efficiency of the present spheres was slightly enhanced compared to the hollow spheres BiFeO_3_ reported previously, representing one of the highest photocatalytic activities among BiFeO_3_ materials to date [9]. The BiFeO_3_ hollow spheres significantly outperformed bulk material and unsupported BiFeO_3_ nanoparticles [37,51]. In agreement with data found in the literature, their hollow morphology enhanced mass transfer rates, facilitating the transport of the dye and degradation products to and from the catalyst surface [10,14]. Additionally, enhanced light scattering, internal reflection, and light trapping within the cavities improved their light utilization compared to non-hollow analogs. The thin walls of the hollow spheres also reduce the charge-carrier lengths, decreasing electron–hole recombination before excited electrons reach the catalyst surface [10].

The kinetics of the photodegradation reaction can be described by the Langmuir–Hinshelwood kinetics model according to Equation (1):

(1) $$r=-\frac{dc}{dt}=\frac{k_{r}K_{c}}{1+K_{c}}$$

where *r* (mg L^−^^1^ min_−__1_), *k_r_* (mg L^−^^1^ min^−^^1^), *K_c_* (L min^−^^1^), *c* (mg L^−^^1^), and *t* (min) stand for the reaction rate, reaction rate constant, adsorption coefficient of the reactant, reactant concentration, and irradiation time, respectively. After simplifications, the Langmuir–Hinshelwood model can be expressed for diluted systems as shown in Equation (2), where C0 is the initial concentration of Rhodamine B at time *t* = 0, *C* represents the concentrations at different irradiation times *t*, and *k* is the pseudo-first-order rate constant of photodegradation (min^−1^). The obtained rate constant of *k* = 2.7 × 10^−^^2^ min^−^^1^ confirms the enhanced photocatalytic properties of the synthesized BiFeO_3_ hollow spheres [54].

(2) $$\ln\left(\frac{C}{C0}\right)=-kt$$

Furthermore, we evaluated the impact of catalyst concentration on degradation reactions by varying the catalyst concentrations from 0 to 3 g/L (see Figure 7c). At lower concentrations, an insufficient amount of BiFeO_3_ hollow spheres, i.e., active catalyst sites, resulted in a significant drop in the removal percentages. With increasing concentration of the photocatalyst, we found a range of high photocatalytic efficiency (1.0–2.0 g/L), with an optimal concentration between 1.25 and 1.50 g/L achieving complete degradation of the organic pollutant within 2 h. At concentrations above 1.75 g/L, the degradation of Rhodamine B decreased as a result of the increased opacity of the reaction mixture, with diminished light penetration and, consequently, a decrease in overall removal efficiency [9,51,54].

**Figure 8:**
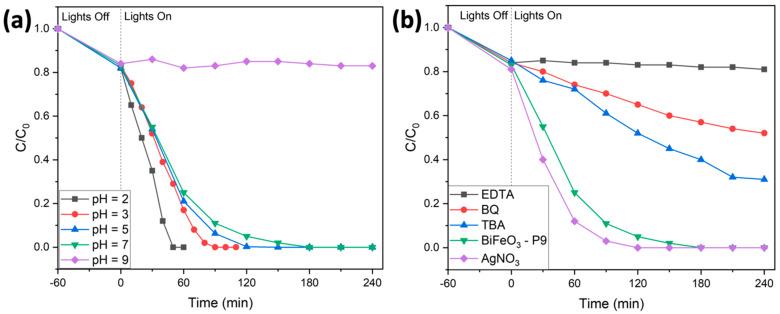
(**a**) Influence of pH on the degradation of Rhodamine B. (**b**) Trapping experiments in the photodegradation of Rhodamine B using BiFeO_3_ hollow spheres.

The stability and reusability of the synthesized BiFeO_3_ hollow spheres were tested in photocatalytic degradation reactions of Rhodamine B, as illustrated in Figure 7d. After each complete catalytic reaction, the BiFeO_3_ photocatalyst was recovered from the reaction mixture by centrifugation, and then thoroughly washed with water and ethanol. After drying, the powder was reused in subsequent catalytic experiments with fresh Rhodamine B solution. The photocatalytic performance was nearly identical in all four consecutive runs, confirming the overall stability and reusability of the synthesized BiFeO_3_ hollow spheres. The stability of the recovered catalyst was further validated by its XRD diffractogram, as shown in Figure 3b. The diffractogram after four catalytic runs remained nearly identical to that of the catalyst before its use in the photodegradation reactions, with no detected impurities (such as Bi_2_O_3_ or Bi_2_Fe_4_O_9_). To further optimize the reaction conditions in terms of complete and fast degradation of Rhodamine B, we varied the pH of the reaction mixture between 2 and 9, as shown in Figure 8a. At pH levels above 7, both the adsorption and degradation of Rhodamine B were less effective compared to neutral or acidic conditions. This can be explained by the presence of negatively charged hydroxyl anions on the catalyst surface, which repelled the negatively charged carboxylate groups in Rhodamine B. For pH values below the pKS2 of Rhodamine B (pKS2 = 3.22), the carboxylic group of Rhodamine B remains in its protonated form, resulting in an enhancement of the degradation efficiency [54]. For instance, at pH 2, the dye was completely removed from the solution in approximately 45 min, as depicted in Figure 8a.

Furthermore, we investigated the reaction mechanism of the photodegradation process through standard trapping experiments using different radical scavengers (see Figure 8b). During the degradation reaction, we observed a blueshift in the maximum absorbance peak from 553 nm to 548 nm. This shift was attributed to the formation of intermediate species, resulting from the initial removal of ethyl groups and the breaking of the conjugated chromophore [54]. The impact of various scavengers on the photodegradation process was investigated by adding typical radical scavengers and observing their effects on the dye removal efficiency. We used tert-butyl alcohol (TBA, 2 mM) to scavenge hydroxyl radicals (∙OH), benzoquinone (BQ, 0.5 mM) to scavenge superoxide radicals, and ethylenediaminetetraacetic acid (EDTA, 2 mM) to scavenge holes. The results showed significant decreases in dye removal efficiency: a 31% reduction with TBA, a 52% reduction with BQ, and an 81% reduction with EDTA.

These findings suggest that hydroxyl radicals, superoxide radicals, and photogenerated holes all play crucial roles in the degradation process. Additionally, the introduction of the electron scavenger AgNO_3_ led to a slight improvement in removal efficiency, as shown by the complete removal of Rhodamine B in approximately 120 min. This enhancement is likely due to the consumption of excited electrons, which improves electron–hole separation and reduces recombination rates. This indicates that effective electron–hole separation is also vital for efficient photodegradation [54]. Overall, the mechanism of the photodegradation aligns with the one that we previously described [9].

The synthesized BiFeO_3_ hollow spheres were then applied as photocatalysts in the degradation of the emerging pollutant metronidazole (MNZ, 2-methyl-5-nitroimidazole-1-ethanol) under visible-light irradiation using 427 nm and 440 nm Kessil lights. It should be noted that metronidazole is a nitroimidazole antibiotic extensively used to treat infections caused by anaerobic bacteria and various protozoans. Additionally, it is also used as a supplement in fish and poultry feed to promote weight gain [66,67,68,69]. Residual concentrations of MNZ in surface waters and wastewater typically range from 1 to 10 ng/L. Due to its high solubility and non-biodegradable nature, MNZ tends to accumulate in aquatic environments. The presence of MNZ in water systems is a significant concern due to its toxicity, potential mutagenicity, and carcinogenicity [69]. Therefore, the effective elimination of MNZ from water systems is crucial for protecting both environmental and human health.

**Figure 9:**
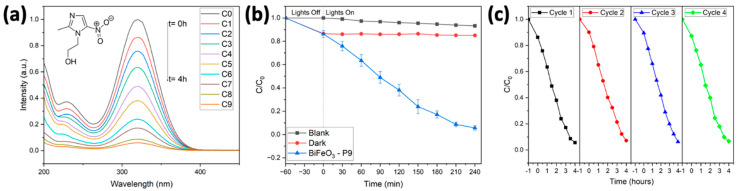
Photocatalytic degradation of metronidazole using BiFeO_3_ hollow spheres (sample P9): (**a**) UV spectra of metronidazole during the degradation reaction. (**b**) Removal of metronidazole as a function of irradiation time. (**c**) Reuse of P9 in consecutive degradation reactions.

Figure 9 illustrates the UV–Vis absorption spectra of a metronidazole (MNZ) solution undergoing photocatalytic degradation using BiFeO_3_ hollow spheres (sample P9) at pH = 7. The spectra display a characteristic absorption peak at 320 nm. In the absence of a photocatalyst, the concentration of metronidazole remained unchanged for a total of 180 min. In the presence of the photocatalyst, the adsorption efficiency of MNZ in the dark was found to be 15%, indicating that the hollow spheres possess similar adsorption capacities for both MNZ and Rhodamine B. As the irradiation time increased, the intensity of the maximum absorbance peak gradually decreased, disappearing after 240 min, indicating the total degradation of metronidazole. This demonstrates the high efficiency of BiFeO_3_ hollow spheres as a photocatalyst for the degradation of metronidazole.

The stability of the BiFeO_3_ photocatalyst was assessed through recycling experiments. The photocatalytic performance of the hollow spheres for each run is depicted in Figure 9c. After four recycling runs, the photocatalytic properties were retained, as no significant loss in photocatalytic activity was observed. This underscores the high stability and reusability of the BiFeO_3_ hollow spheres as a photocatalyst for the degradation of organic pollutants through visible-light photocatalysis.

## 3. Materials and Methods

### 3.1. Characterization Techniques and Equipment

The structure and phase purity of the synthesized materials were characterized using a Bruker D2 Phaser X-ray diffractometer with a 1.54184 Å copper tube. Utilizing the DIFFRACC.EVA V4.3.1.2 software, a semi-quantitative analysis of the diffraction pattern was performed to identify secondary phases. The morphological analysis of the sample was conducted using scanning electron microscopy (SEM) and energy-dispersive X-ray spectroscopy (EDX). A MIRA 3 field-emission electron microscope from TESCAN, equipped with a Bruker X-Flash 6–30 detector with a resolution of 123 eV in Mn Kα, was used for this purpose. Inductively coupled plasma optical emission spectroscopy measurements were performed on a Thermo Scientific iCAP 7400 ICP-OES spectrometer (Thermo Scientific, Waltham, MA, USA). Calibration curves were constructed from a multi-element standard solution 6 for ICP, grade Trace CERT (Sigma Aldrich, St. Louis, MO, USA) (100 mg/L). The detection and quantification limits were calculated by analyzing blank samples with at least 8 replicates and multiplying the standard deviation by 3 to obtain the limit of detection (LD) and by 10 to obtain the limit of quantification (LQ). Quality control metal element analysis was conducted by employing certified reference material (NIST3118A, Fe Certipur, SRM 3106) every 10 samples. The adsorption–desorption isotherms were recorded on a Quantachrome Autosorb IQ 6AG/HOB analyzer (Boynton Beach, FL, USA). The Brunauer–Emmett–Teller (BET) equation was utilized to determine the specific surface areas, while the Barrett–Joyner–Halenda (BJH) algorithm was employed to derive the pore size distribution from the head adsorption branches of the isotherms. The diffuse reflectance spectrum was measured by UV–Vis spectroscopy (PerkinElmer, Waltham, MA, USA), with a wavelength range of 200–1000 nm, using an integrating sphere. The Kubelka–Munk transformation was applied to the obtained spectra to determine the band gap values. The X-ray photoelectron spectroscopy (XPS) analysis was performed using a Phi 5000 VersaProbe III (Physical Electronics, Chanhassen, MN, USA), equipped with a hemi-spherical quartz monochromator. The survey scan was conducted at 255 kV, while high-resolution scans were performed at 60 kV. Calibration was achieved using the adventitious carbon peak at 284.8 eV and the Fermi energy level to ensure high accuracy.

### 3.2. Synthesis of BiFeO_3_ Hollow Spheres

All chemical reagents used in these experiments were purchased from Sigma-Aldrich (St. Louis, MO, USA) as analytical grade and used without any further purification. In a typical synthesis, 30 mL of 2-methoxyethanol (C_3_H_8_O_2_) was added to a mixture of 2.06 mmol of bismuth nitrate pentahydrate Bi(NO_3_)_3_·5 H_2_O (molecular weight = 485.07 g/mol), 2 mmol of iron nitrate nonahydrate Fe(NO_3_)_3_·9 H_2_O (molecular weight = 404.00 g/mol) and 2 mmol of citric acid (C_6_H_8_O_7_ molecular weight = 192.12 g/mol). The suspension was acidified with 3 mL of HNO_3_ (2M), and the resulting solution was stirred vigorously overnight. The solvent of the reaction mixture was evaporated in a vented oven at 50 °C. The remaining white precipitate was washed three times with 5 mL of water and then with 3 mL of ethanol at 0 °C. After drying overnight at 80 °C, the powder was dissolved in a mixture of 40 mL of 2-methoxyethanol and 5 mL of HNO_3_ (2M). The reaction mixture was stirred vigorously at room temperature, and a solution of 1 g of Pluronic P123 (PEG-PPG-PEG, poly(ethylene glycol)-block-poly(propylene glycol)-blockpoly(ethylene glycol), molecular weight ~5800 g/mol) in 10 mL of 2-methoxyethanol was added dropwise. The reaction mixture was stirred overnight at room temperature and then transferred into a plastic container with a narrow mouth. The solvent was evaporated slowly at 40 °C, and the precipitate was collected and dried in a ventilated oven at 80 °C overnight. Finally, the precursor was calcined at 500 °C for 1 h, with an intermediate ramp at 200 °C for 2 h (heating rate = 1 °C).

### 3.3. Photocatalytic Experiments

We assessed the photocatalytic activity of the BiFeO_3_ samples at room temperature using Rhodamine B as a model dye, with an initial concentration of 5 mg/L at pH 7. In a standard experiment, 50 mg of the BiFeO_3_ sample was added to 50 mL of the dye solution. The mixture was stirred in darkness for 60 min to establish adsorption–desorption equilibrium between the catalyst and the dye. Subsequently, the samples were irradiated using four Kessil lamps—two emitting at 427 nm (PR160-427 nm) and two at 440 nm (PR160-440 nm)—positioned exactly 10 cm from the center of the reaction mixture. Every 30 min, the catalysts were separated from the mixture via centrifugation at 1000 rpm for 3 min. The dye concentration over time was determined by measuring the absorbance at the maximum intensity of the absorption peak using the Lambert–Beer equation.

For the reusability study, the catalyst was isolated through centrifugation, washed repeatedly with water and ethanol, and then dried overnight at 80 °C. The catalyst was subsequently reused in consecutive photocatalysis experiments, following the same procedure as described above.

The absorption spectrum of Rhodamine B was measured using a GENESYS 30TM UV–Vis spectrophotometer with a tungsten–halogen light source and silicon photodiode detector. The spectra were fitted with the Thermo Scientific VISIONlite PC software suite (Version 5.0).

## 4. Conclusions

In summary, monodisperse bismuth ferrite hollow spheres were synthesized by a facile, novel, two-step, evaporation-induced self-assembly (EISA) approach. The direct reaction of the corresponding metal nitrates with citric acid in the presence of the block copolymer Pluronic P123 in water and ethanol led to the formation of predominantly bulk material and minor amounts of BiFeO_3_ hollow spheres during the evaporation-induced self-assembly process. X-ray diffraction studies showed that additional phases such as Bi_2_O_3_ were also formed during the reaction. The concentration of the structure-directing agent P123 and doping the semiconductor with 5% gadolinium did not impact the phase purity or morphology of the synthesized material. Impurities arose due to the rapid hydrolysis of bismuth nitrate in water. Using prefabricated Bi/Fe citrate complexes in methoxyethanol with P123 as the structure-directing agent resulted in the formation of monodisperse BiFeO_3_ hollow spheres with an average diameter of approximately 400 nm during the EISA process. XRD measurements confirmed the synthesis of phase-pure BiFeO_3_ hollow spheres, characterized by a rhombohedral perovskite phase with R3c symmetry. Diffuse reflectance UV–Vis spectra revealed that the BiFeO_3_ hollow spheres absorb a significant amount of light in the visible region and have a band gap of 2.12 eV. The spheres were utilized in photocatalytic degradation experiments with the dye Rhodamine B and the antibiotic metronidazole as the model organic pollutants under visible-light irradiation (427 nm and 440 nm). The BiFeO_3_ hollow spheres exhibited exceptionally high photocatalytic activity in both cases, achieving complete dye degradation in approximately 140 *min* and complete antibiotic degradation in approximately 240 min. In the case of Rhodamine B degradation, faster degradation was achieved by slightly increasing the catalyst concentration or reducing the pH. Additionally, catalyst recycling experiments demonstrated in both cases that the semiconductor spheres are stable under the applied reaction conditions and can be successfully recycled without any loss in photocatalytic performance. The enhanced photodegradation activity of the BiFeO_3_ catalysts can be attributed to their hollow sphere morphology. Trapping experiments indicated that photogenerated holes (h+), hydroxyl radicals (·OH), and superoxide radicals (·O_2_^−^) are the primary active species in the photodegradation process.

## Figures and Tables

**Table 1:** Experimentally obtained and theoretical doping contents, as well as differences in Pluronic P123 concentration.

Sample	Pluronic 123 Conc. in mmol	Doping Content– Molar Ratio (%)	
		Experimental Values by ICP-OES	Calculated Values
P1	2.72	0	0
P2	3.06	-	0
P3	3.60	-	0
P4	4.08	-	0
P5	2.72	4.98	5.00
P6	3.06	4.95	5.00
P7	3.60	4.95	5.00
P8	4.08	4.96	5.00

## Data Availability

The original contributions presented in the study are included in the article (and Supplementary Materials), further inquiries can be directed to the corresponding authors.

## Supplementary Materials

The following supporting information can be downloaded at https://www.mdpi.com/article/10.3390/molecules29153592/s1, Figure S1: EDS analysis of BiFeO_3_ hollow spheres/bulk; Figure S2: N_2_ adsorption–desorption isotherm and pore size distribution (inset) of BiFeO_3_ hollow spheres (P9); Figure S3: UV–Vis reflectance spectra of P9 and Kubelka–Munk plot.
