# Supplementary material for: Enhanced Visible-Light Photocatalytic Activity of Bismuth Ferrite Hollow Spheres Synthesized via Evaporation-Induced Self-Assembly
Source: Molecules. 2024 Jul 30;29(15):3592. doi: 10.3390/molecules29153592 (PMC11314036; doi:10.3390/molecules29153592)
Supplement: Supplementary file 1 [file molecules-29-03592-s001.zip › molecules-3114544-supplementary.pdf]

molecules

Supporting Information

Enhanced Visible-Light Photocatalytic Activity of Bismuth Ferrite Hollow Spheres Synthesized via Evaporation-Induced Self-Assembly

Thomas Cadenbach [1,*], Valeria Sanchez [2], Karla Vizuete [3], Alexis Debut [3], Carlos Reinoso [4] and Maria J. Benitez [2,*]

[1] Departamento de Ingeniería Ambiental, Instituto de Energía y Materiales, Colegio Politécnico de Ciencias e Ingenierias, Universidad San Francisco de Quito, Quito 170901, Ecuador
[2] Departamento de Física, Facultad de Ciencias, Escuela Politécnica Nacional, Ladrón de Guevara E11-253, Quito 170517, Ecuador
[3] Centro de Nanociencia y Nanotecnología, Universidad de las Fuerzas Armadas ESPE, Av. Gral. Rumiñahui s/n, Sangolquí 171523, Ecuador
[4] School of Physical Sciences and Nanotechnology, Yachay Tech University, Hda. San José s/n y Proyecto Yachay, Urcuqui 100115, Ecuador
* Correspondence: maria.benitezr@epn.edu.ec (M.J.B.); tcadenbach@usfq.edu.ec (T.C.)

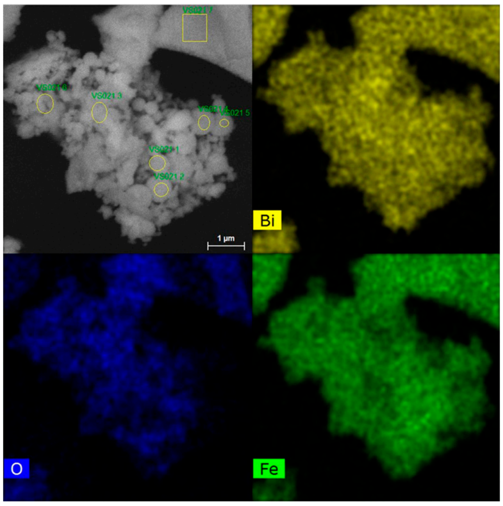

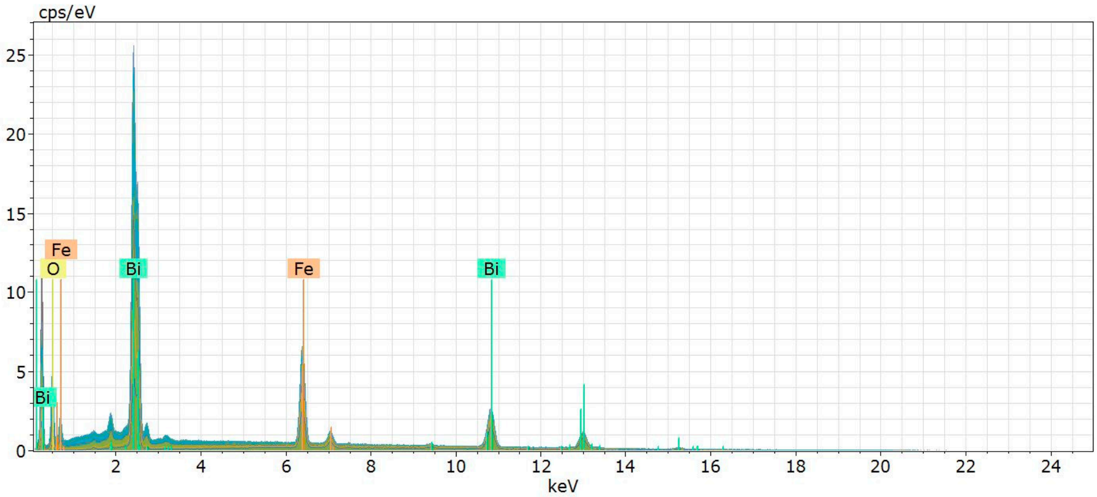

Spectrum	O	Fe	Bi
VS021 1.spx	17,93142	17,66422	67,40436
VS021 2.spx	17,22052	17,07258	66,11069
VS021 3.spx	17,02121	18,01994	66,95885
VS021 4.spx	17,91735	17,97905	66,70921
VS021 5.spx	17,90121	17,24757	66,66231
VS021 6.spx	17,59862	18,09468	67,00367
VS021 7.spx	17,45673	17,87914	66,67518

Figure S1. EDS Analysis of $BiFeO_3$ hollow spheres/bulk.

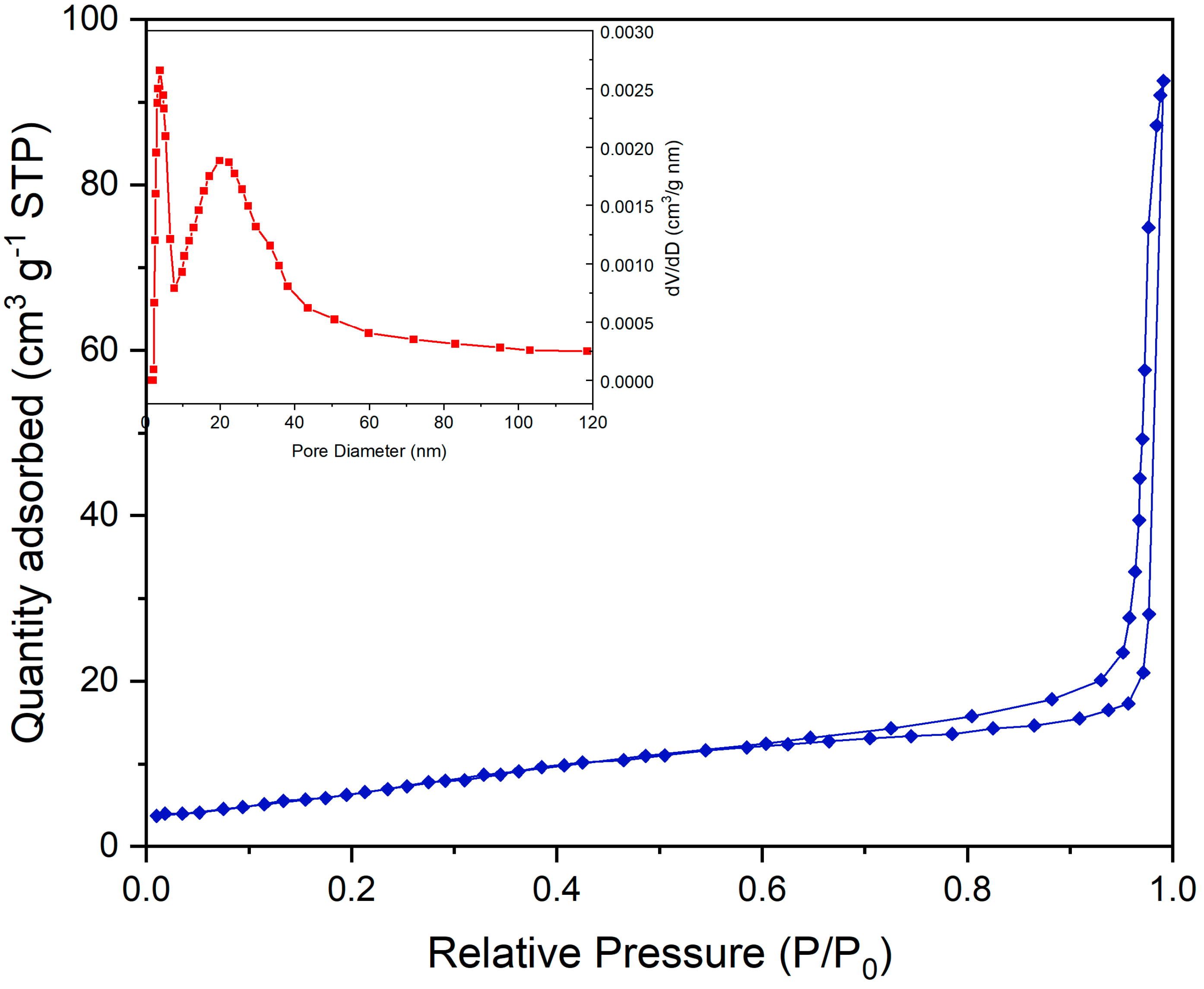

Figure S2. N_2 adsorption–desorption isotherm and pore size distribution (inset) of $BiFeO_3$ hollow spheres (P9).

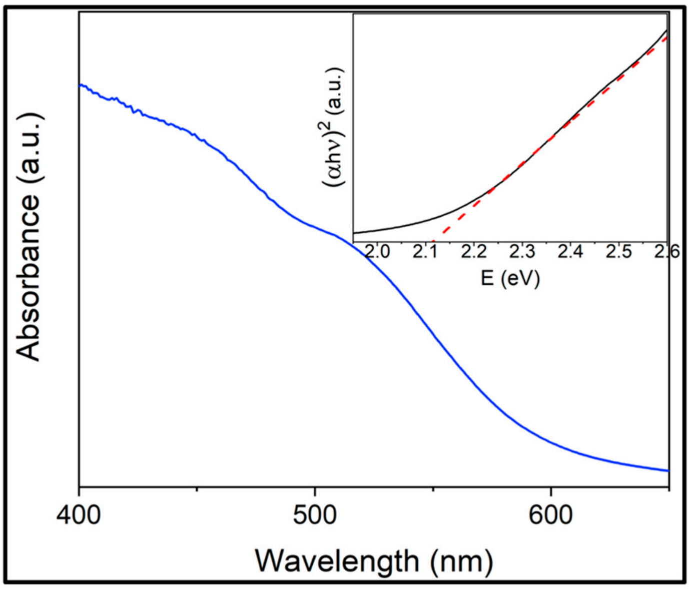

Figure S3. UV-Vis Reflectance spectra of P9. Kubelka-Munk Plot.
